# Structural and Functional Implication of RAP80 ΔGlu81 Mutation

**DOI:** 10.1371/journal.pone.0072707

**Published:** 2013-09-09

**Authors:** Vikrant, Rajan Kumar, Lumbini R. Yadav, Pallavi Nakhwa, Sanjeev K. Waghmare, Peyush Goyal, Ashok K. Varma

**Affiliations:** Advanced Centre for Treatment, Research and Education in Cancer, Kharghar, Navi Mumbai, Maharashtra, India; Department of Biotechnology, CGO Complex, New Delhi, India; George Washington University, United States of America

## Abstract

Receptor Associated Protein 80 (RAP80) is a member of RAP80-BRCA1-CCDC98 complex family and helps in its recruitment to the DNA damage site for effective homologous recombination repair. It encompasses two tandem UIMs (UIM1 and UIM2) motif at its N-terminus, which interact with K-63 linked polyubiquitin chain(s) on H2AX and thereby assemble the RAP80-BRCA1 complex at the damage site. Nevertheless, how RAP80 helps in the structural integrity of BRCA1 complex is still elusive. Considering the role of RAP80 in the recruitment of BRCA1 complex at the DNA damage site, we attempted to explore the molecular mechanism associated with RAP80 and mutation that causes chromosomal aberrations due to its loss of function. There is a significant loss in structural characteristics of RAP80 ΔE81, which impairs its binding affinity with the polyubiquitin chain. This leads to the defective recruitment of RAP80 and BRCA1 complex at the DNA damage site. The results presented here are very useful in understanding the cause of various repair defects (chromosomal aberration) that arise due to this mutation. Comparative study of wild type and ΔE81 could be helpful in designing the small molecules that can potentially compensate the deleterious effect(s) of ΔE81 and hence useful for therapeutic application.

## Introduction

Compromised genomic integrity leads to various genetic disorders and cancer. However, genomic stability is accomplished by the recital action of several cellular events, including DNA replication, DNA repair, senescence and cell death [1]. Cells have evolved a complex, dynamic and highly regulated network to achieve extreme fidelity, called DNA damage response (DDR). In genotoxic stress, DDR coordinates numerous cellular processes like cell cycle regulation, chromatin remodeling, DNA repair and transcription [2]. Sensing of DNA damage and promulgation of the DDR signaling cascade involve recruitment and assembly of many DDR mediators and effectors at the sites of damage [3] [4]. Double strand breaks elicit the activation of ATM and ATR kinases, which phosphorylate histone variant H2AX and MDC1 [5] [6] [7] [8] [9], [10] [3]. This event endorses the assembly of DDR mediators, which in turn facilitate the recruitment of UBC13/RNF8 to the DNA damage sites [11] [12], [13] [14]. In the signaling pathways, eventually this leads to the formation of polyubiquitin chains on H2AX, which are recognized by RAP80 [7], [8], [9] [10]. RAP80 has two tandem UIM (Ubiquitin-Interacting Motif) at its N-terminus, ABRAXAS (CCDC98) Interacting Region (AIR) at the central domain, and two zinc finger domains at its C-terminus [15]. It has been reported that RAP80 forms a stable complex with BRCA1 through an intermediate binding partner CCDC98 [16], [17], [18]. CCDC98 has a consensus sequence SXXF motif at C-terminus, which involves in interaction with BRCA1-BRCT phosphospecific binding domain [16], [18] [19], [20]. BRCA1 acts as a tumor suppressor gene in hereditary breast and ovarian cancer, and plays a diverse role in cell cycle regulation, transcription control and DNA damage repair [21], [22], [23], [24], [25]. C-terminus of BRCA1 (BRCT) is essential for its co-localization with H2AX [26].

RAP80 acts upstream of CCDC98 and BRCA1 in DDR, and is required for the localization of the BRCA1 complex to ionizing radiation (IR)-induced foci (IRIFs) [17], [18], [27]. RAP80 Knockdown cells showed hypersensitivity to IR and ultraviolet (UV) light, cell cycle dysfunction and defective homologous recombination (HR) repair [10], [16], [17], [18]. RAP80 and p53 auto- regulate each other and has influence on apoptosis [28]. Loss of RAP80 alleles (RAP80^−/−^) increase the susceptibility to lymphoma, and promote tumor development in both p53^−/−^ and p53^−/+^ mice [29]. UIM1 and UIM2 motifs of RAP80 are very crucial since deletion of either or both significantly perturb the foci formation of RAP80-BRCA1 complex at the DNA damage site [30].

A novel alteration, c.241–243delGAA (ΔE81) that leads to an inframe deletion of glutamic acid residue has been identified at UIM1 motif of RAP80 [30]. The RAP80 ΔE81 variant was found in a patient diagnosed with breast cancer, and is highly conserved among all the vertebrates. This variant showed an observed frequency of 0.9% (1/112) in the familial cases compared to 0.3% (1/325) in the controls (P¼0.45; OR¼2.92; CI¼0.18–47.1). One RAP80 ΔE81 carrier was also diagnosed with bilateral breast cancer in a group of 503 breast cancer cases (0.2%, 1/503). RAP80 ΔE81 expressing cells showed abrogation of DSB localization of the RAP80–BRCA1 complex and exhibited genomic instability (chromosomal aberration) [30]. In this study, we have carried out a comparative structural, stability and binding analysis of RAP80 (1–130) wild type (referred as RAP80 wild type or wild type henceforth) and RAP80 (1–130) ΔE81 (referred as RAP80 ΔE81 or ΔE81 henceforth) to understand the functional implication(s) of this mutation. To our knowledge, this is the first multi model approach combining *in-silico* and *in-vitro* methods to study the functional implications of RAP80 wild type and the ΔE81. RAP80 ΔE81 relatively exhibited less thermal stability and significant secondary structure distortion, which impaired its binding affinity with di (poly)-ubiquitin. This further leads to defective recruitment of RAP80-BRCA1 complex to the DNA damage site and subsequently giving rise to genomic instability. Our study will be helpful in understanding the role of UIM motifs of RAP80 in RAP80-BRCA1 complex recruitment and hence their DNA damage repair function. It will further assist in elucidation of mechanism that alters the binding affinity of RAP80 UIMs for polyubiquitin chain due to ΔE81 mutation, and thereby its implication on damage repair.

## Results and Discussion

RAP80 is 80 KDa nuclear protein that interacts with retinoid-related testis-associated receptor [15]. It is a member of BRCA1 complex and facilitates the recruitment of BRCA1 to the DNA damage site. Thus, it is a multifunctional molecule that plays a dispersive role in steroid hormone signaling, and BRCA1 mediated homologous recombination repair. SiRNA mediated silencing, and knockout studies of RAP80 showed defective recruitment of BRCA1 complex and hence the perturbed DNA repair [29], [31], [32], [33]. *In-vitro* and *in-silico* findings from our study, will be helpful in understanding the mutational consequence of RAP80 ΔE81 in DNA damage and repair pathway. To our knowledge, this is the first report on a comparative functional characterization of RAP80 wild type and ΔE81.

### Structural Organization of RAP80

**Figure 1:**
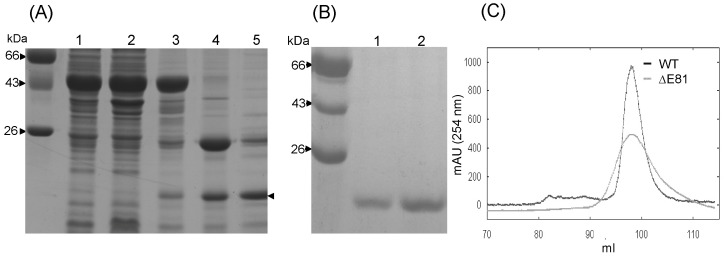
Expression and purification profile of RAP80 wild type and ΔE81. (**A**) Whole-cell lysate, and supernatant obtained after sonication and centrifugation were heated with Laemmli buffer and loaded onto SDS-PAGE. Similarly, protein was eluted from beads by heating with Laemmli buffer and loaded on gel. Lane 1-Total protein, 2-soluble protein, 3-fusion protein bound on beads, 4- protein after on beads cleavage, 5-elution fraction of affinity purified proteins. Single arrow - RAP80 wild type protein (**B**) Purified protein after gel filtration chromatography on SDS-PAGE. Lane 1- RAP80 ΔE81, 2- RAP80 wild type (**C**) Overlay of gel filtration spectra of RAP80 wild type and ΔE81 (Superdex 200). Elution profiles of both the protein were similar and suggest their monomeric nature.

Coomassie stained SDS-PAGE for RAP80 wild type and ΔE81 showed a single band corresponding to 14 KDa (**Figure 1A, B**). A single peak spectrum was observed in size exclusion chromatography (**Figure 1C**). Purified proteins were further subjected to MALDI-TOF (Matrix Assisted Laser Desorption Ionization -Time of Flight), and spectra corresponding to 14.958 KDa and 14.815 KDa for RAP80 wild type and ΔE81 respectively, were recorded with greater sensitivity. We found a close match between experimentally derived (wild type: 14.958 KDa, ΔE81 14.815 KDa) and theoretically predicted molecular weight (wild type: 14.898 KDa, ΔE81 14.751 KDa) (**Table 1**). The presence of single peak in mass spectroscopy and size exclusion chromatography indicates monomeric behavior of RAP80 wild type and ΔE81 (**Figure 1C**).

**Table 1:** Molecular weight estimation of purified protein.

	Theoretical Mol. Wt. (kDa)^a^	Ve/Vo^b^	Experimental Derived Mol. Wt. (kDa)	
			Gel Filtration Chromatography	Mass spectrometry (MALDI-TOF)
Wild type	14.897	2.107527	15.8	14.9
ΔE81	14.750	2.107527	15.8	14.8

Ve/Vo: Elution volume/Void volume ratio in gel filtration chromatography (superdex 200 16/60).

a Determined from Protparam, Expasy.

b Determined from standard myoglobin, ovalbumin, albumin, IgG, Ferritin.

**Figure 2:**
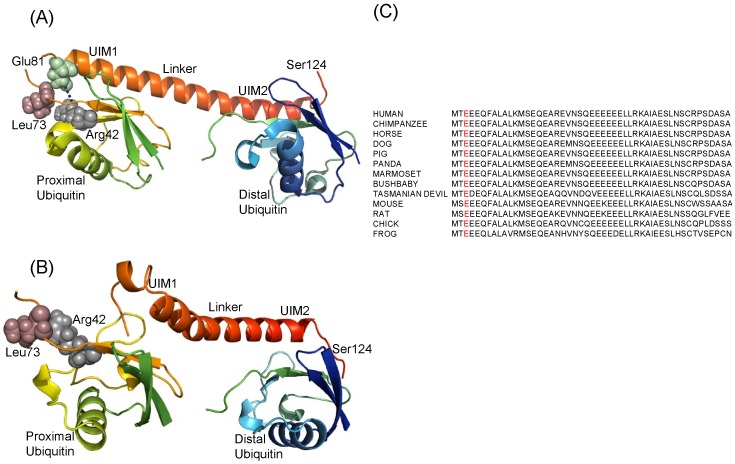
Binding interaction of RAP80 UIMs and ΔE81 with Di-Ub (K-63 linked). (**A**) Structure of Di-Ub (K-63 linked)-RAP80 UIMs (79–124) wild type (PDB ID: 2RR9), and (**B**) Di-Ub (K-63 linked)-RAP80 (79–124) UIMs ΔE81. Wild type and Di-Ub (K-63 linked) complex is stabilized by weak intermolecular interactions. α-helix of RAP80 (79–124) UIM ΔE81 was found to be distorted. (**C**) multiple sequence alignment of UIMs region showed it's highly conserved nature in various species. Glu 81 residue is highlighted in red color.

RAP80 (79–124) UIMs ΔE81 structure was successfully modeled using protein modeler [34], [35] with a acceptable Ramachandran plot [36] [37]. UIM1 and UIM2 are connected with a linker in a head to tail manner. The three-dimensional structure of wild -type looks overall 59 Å long and α-helical in nature. However, in case of mutant, α–helix is partly distorted and shorten to 45 Å. UIM1 and UIM2 bind with their respective proximal and distal ubiquitin of Di-Ub (K-63 linked) in 1∶1 affinity ratio [38] [39]. Glu residue at 81 position was found to be highly conserved (Figure 2C) and forms ionic bond and hydrophobic interaction, with the Arg42 and Leu73 residue of proximal ubiquitin, respectively. It is widely reported that hydrogen bonding and hydrophobic interactions play an important role in protein stability and selection of the specific target [40]. There are changes in weak intermolecular interactions between RAP80 UIMs, RAP80 UIMs ΔE81 and Di-Ub (K-63 linked) (**Figure 2A, B**). The hydrogen bonds between Gln84, Ser92, Glu95, Ser117, Gln102 residues of RAP80 UIMs and the Leu8, Gly47, Thr66, His68, Arg72 of ubiquitin, and the hydrophobic interactions between Ser 92, Ser 117 of RAP80 UIMs and Ile44, Phe45, Ala46, Gly47, His68 of proximal ubiquitin are stabilizing the binding interface. However, a drastic conformational change in RAP80 UIMs ΔE81 was observed which significantly alter the weak intermolecular interactions with ubiquitin. Met 79, Glu 83 and Glu 93 of UIMs are involved in hydrogen bonding with His 68, Gly 47 of ubiquitin. Hydrophobic interactions between the Met 79, Arg122, residues of RAP80 UIMs ΔE81 with the Phe4, Leu43, Ile44, Phe45, Gly47, Lys48, Gln49, Leu50, Glu64, Ser65, Thr66, His68 residues of ubiquitin primarily holds the complex. Structural distortion in RAP80 UIMs ΔE81 probably renders its binding interaction unfavorable with Di-Ub (K-63 linked).

**Figure 3:**
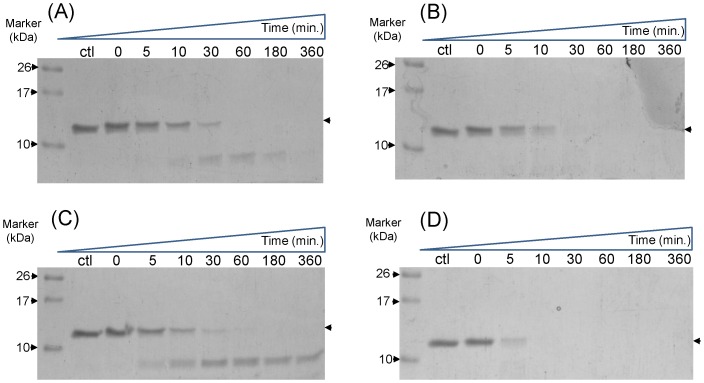
Resistivity profile of RAP80 wild type and ΔE81 towards Protease digestion. Limited proteolysis of RAP80 wild type (**A, C**) and ΔE81 (**B, D**) using trypsin (**A, B**) and Chymotrypsin (**C, D**) as proteases. Wild type showed relatively high resistance towards proteolysis as indicated by less rate of decrease of band intensity. This suggests a well-folded structure of wild type compared to ΔE81. Ctl- control was taken as untreated with proteases.

**Figure 4:**
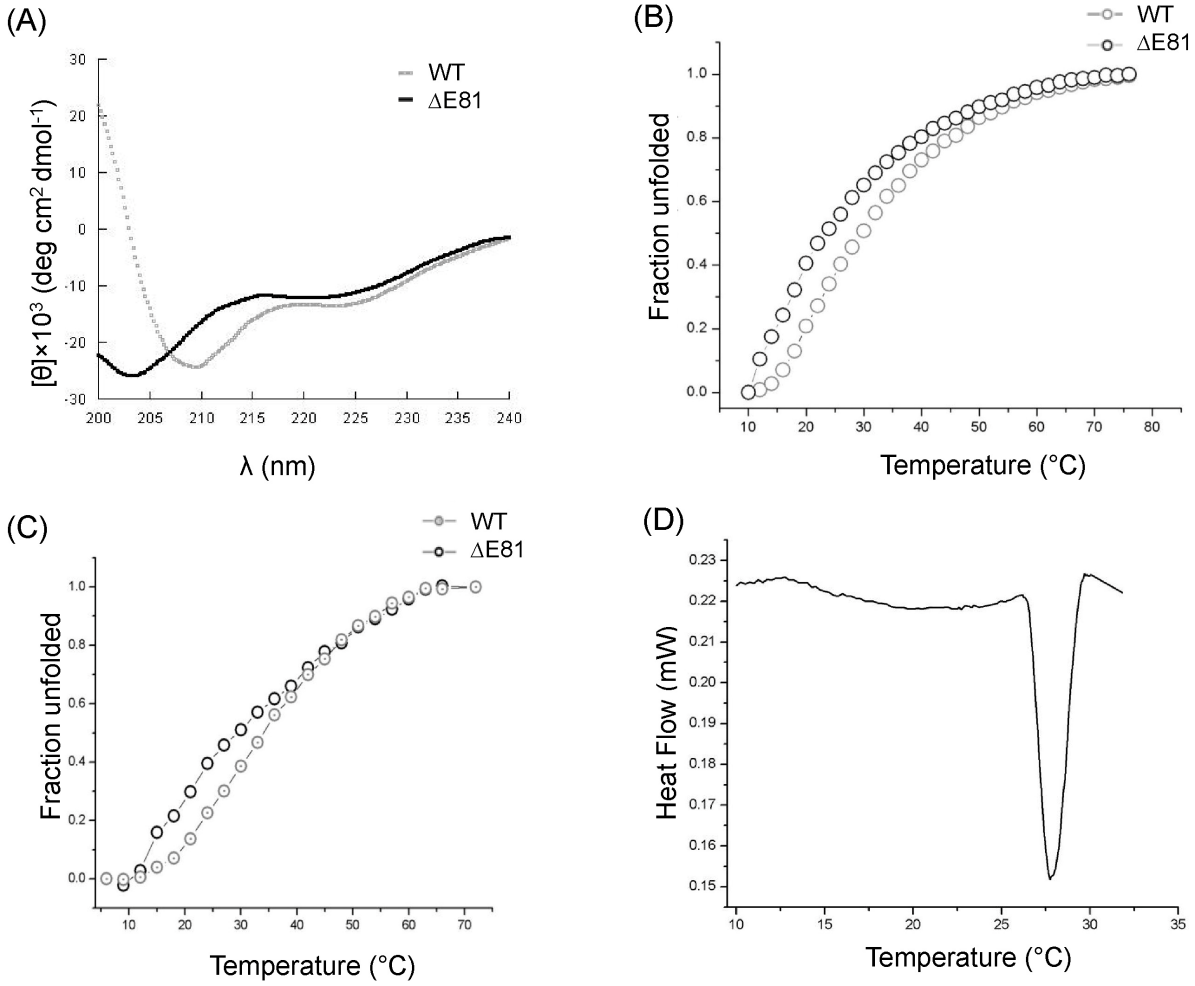
Structure and stability analysis of RAP80 wild type and ΔE81. Secondary structural components and thermal stability of RAP80 wild type and ΔE81. (**A**) Overlay of Far-UV Circular Dichroism spectrum of wild type and ΔE81. Wild-type showed well-defined α/β characteristics compared to a random structure pattern of ΔE81. Thermal stability of RAP80 wild type. (**B**) Thermal denaturation of RAP80 wild type and ΔE81 using Circular Dichroism and (**C**) using ANS as extrinsic fluorophore in Fluorescence. Unfolded fractions were calculated and plotted against different temperatures. (**D**) Differential Scanning Calorimetry profile of RAP80 wild type. Protein showed a well-defined transition around 28°C.

To understand structural integrity and determine the resistivity of RAP80 wild type and ΔE81 against the protease digestion, limited trypsin and chymotrypsin proteolysis was performed. RAP80 wild type and ΔE81 were treated with same concentration of proteases for limited time (**Figure 3A, 3B, 3C, 3D**). RAP80 wild type resistance against protease digestion gives the indication of having a relatively stable domain and well-formed structure. However, susceptibility of RAP80 ΔE81 towards protease digestion suggests that deletion of E81 is responsible for destabilizing the structural integrity of RAP80. Furthermore, we have compared the changes in secondary structure using far-UV circular Dichroism (**Figure 4A**). It was observed that RAP80 wild type has well-defined α/β characteristics whereas structure of ΔE81 showed deviation from typical α/β characteristic to random structure. Earlier report suggests that UIMs motif of RAP80 is found in equilibrium between α-helix and random structure [41]. ΔE81 mutation probably alters the α-helical conformation of RAP80 UIMs which leads to shift the equilibrium towards a random structure pattern.

### Thermal stability

Stability profiles of RAP80 wild type and ΔE81 was compared at secondary (CD) and tertiary (Fluorescence) structure levels. The spectra obtained from Circular Dichroism corresponding to λ at 218 nm showed the maximum change in ellipticity and high signal to noise ratio (**Figure 4B**). Thermal stability of RAP80 ΔE81 (Tm 22°C, ΔG°_H2O_ 1.3±0.2 Kcal/mol, ΔH 1.0±0.5 Kcal/mol) was found significantly low compared to wild type (Tm 29°C, ΔG°_H2O_ 2.0±0.5 Kcal/mol, ΔH 5.0±2.0 Kcal/mol). ANS (8-Anilinonaphthalene-1-sulfonate) fluorescence spectroscopy has an agreement with CD data, and derived Tm value was 23°C for ΔE81 (ΔG°_H2O_ 1.4±0.3 Kcal/mol, ΔH 1.1±0.5 Kcal/mol) and 30°C for RAP80 wild type (ΔG°_H2O_ 2.4±0.5 Kcal/mol, ΔH 8.0±1.1 Kcal/mol) (**Figure 4C**). Both the methods showed that protein most likely unfolds without any intermediate species. These findings were further supported by Differential Scanning Calorimetry, which gave a Tm value of 28°C for RAP80 wild type (**Figure 4D**). However, we could not obtain a defined transition for ΔE81, due to lesser stability and saturation concentration (**Table 2**). These results suggest that three-dimensional folding of RAP80 ΔE81 is impaired in comparison to wild type. These findings also support the helix to random structure transition of UIMs motif. ΔE81 mutation probably shifts this transition equilibrium towards the random structure.

**Table 2:** Thermal parameters of protein unfolding.

Method	Protein	T_m_ (°C)	ΔG°_H2O_ (Kcal/mol)	ΔH (Kcal/mol)
DSC	Wild type	28	-	8.7±1.0
Fluorescence	Wild type	23	2.4±0.5	8.0±1.1
	ΔE81	30	1.4±0.3	1.1+0.5
CD	Wild type	29	2.0±0.5	5.0±2.0
	ΔE81	22	1.3±0.2	1.0±0.5

T_m_ Melting Temperature.

### Binding interaction of RAP80 wild type and ΔE81 with di-Ub (K-63 linked)

**Figure 5:**
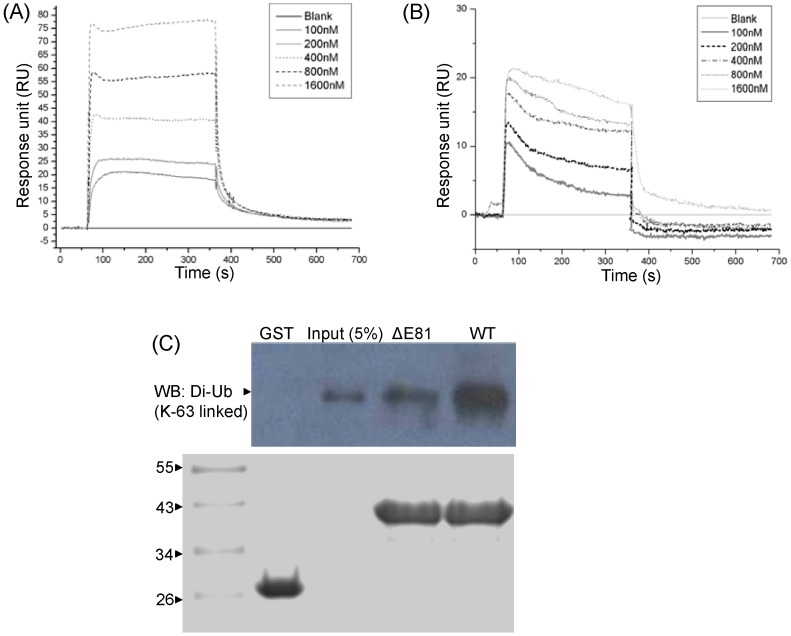
Binding analysis of RAP80 wild type and ΔE81 with Di-Ub (K-63 linked). Sensogram of RAP80 wild type (**A**) and ΔE81 (**B**) determined by Surface Plasma Resonance. 5 µg of ligand {Di-Ub (K-63 linked)} was immobilized on CM5 sensor chip and different concentrations of analytes (wild type and ΔE81) were passed. (**C**) GST pull down assay followed by western blotting. GST-RAP80 wild type and ΔE81were used as a bait and Di-Ub (K-63 linked) as prey. Di-Ub (K-63 linked) was probed with anti-ubiquitin antibody. Ponceau stained PVDF membrane showing the GST and GST fusion protein as bait(s). Wild type showed high binding affinity compare to ΔE81. GST was taken as control.

It is well reported that RAP80 UIMs bind with K-63 linked polyubiquitin chain(s) on the H2AX and recruit the RAP80-BRCA1 complex to the DNA damage site [18] [27]. Considering structural distortion and stability of RAP80 ΔE81, it can be suspected that it would further impair binding affinity for polyubiquitin chain. Binding analysis between RAP80 wild type and ΔE81 with Di-Ub (K-63 linked) has been performed using Surface Plasma Resonance (SPR) and GST pull down assay. The observed binding affinity for RAP80 ΔE81 (K_D_: 0.459 µM) was several fold less as compared to wild type (K_D_: 36.5 nM) in SPR (**Figure 5 A, 5B**). Association rate constant of RAP80 ΔE81 was found significantly lower (Ka: 4.306e^1^M^−1^s^−1^) than wild type (Ka: 3.06e^5^M^−1^s^−1^). Besides this, RAP80 ΔE81 showed high dissociation rate as compared to wild type. Furthermore, association constant of wild type is higher than ΔE81 {K_A (Wild Type)_: 2.74e7 M^−1^, K_A (ΔE81)_: 2.18e^6^ M^−1^}. GST pull down assay also supported the finding obtained using SPR (**Figure 5C**). It can be concluded that RAP80 wild type has higher binding affinity for the polyubiquitin chain, besides, it associates faster than ΔE81. Mutant protein complex {ΔE81-Di (Ub)}was likely unstable due to high dissociation rate and less binding affinity. Alteration in binding affinity of RAP80 ΔE81 could be due to its structural deformation.

## Conclusion

**Figure 6:**
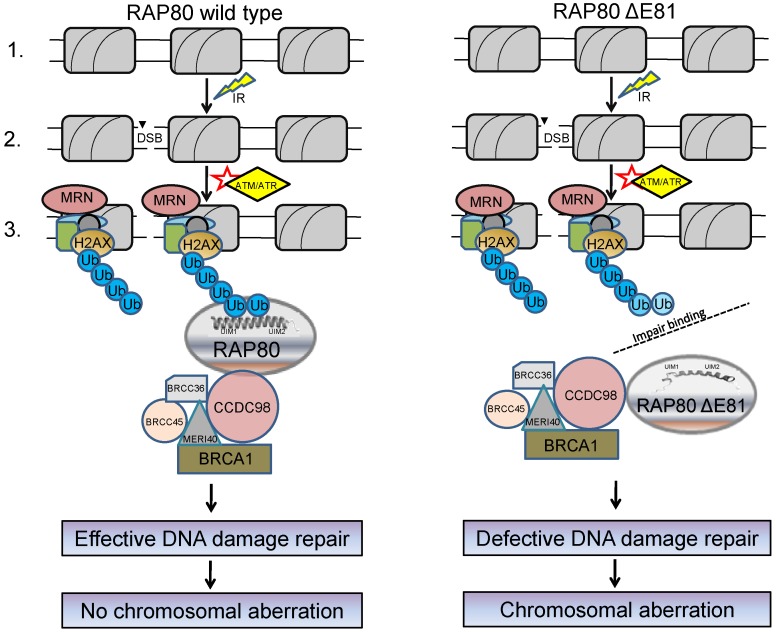
Anticipated mechanism of consequence due to RAP80 ΔE81. The model elucidate a possible mechanism of chromosomal aberration due to RAP80 ΔE81 mutation. In the wild-type RAP80: **Step1**, showed the intact nucleosome complex; **Step 2**, double strand break due to ionization radiation; **Step 3**, ATM/ATR kinase activation and assembly of various damage repair proteins at DNA double strand break (DSB) site followed by formation of polyubiquitin chain(s) on histone(s) (H2AX). The polyubiquitin chain(s) are recognized by RAP80 UIMs motif thereby recruiting the BRCA1 complex to the DNA damage site. However, in case of ΔE81 mutation, interaction between polyubiquitin chain and RAP80 UIM altered due to structural distortion in α-helix which further leads to defective recruitment of the BRCA1 complex. Error-prone DNA damage repair increases the chances of chromosomal aberration and hence the tumorigenesis.

RAP80 wild type and ΔE81 are moderately soluble. Thermal and proteolytic stability of wild type was found significantly higher as compared to ΔE81, but both unfold likely with two state irreversible transition. RAP80 UIMs are found in equilibrium between random-coil and helical states. This fact is supported by low Tm values of both wild type and ΔE81. The reason behind dynamic nature of UIMs is to provide immense flexibility of dissociation and association of ubiquitin molecules during the protein trafficking process. Perhaps UIMs also use this mechanism for multiple mode of binding (monovalent and multivalent) so as to achieve cooperativity in binding interactions. This dynamic nature is essential for a flexible and transient initiation mechanism of the DNA damage repair process. Deletion of 81E residue perhaps alters the helical state conformation, thus shifting equilibrium towards a random structure. Helical to random structure transition results in loss of several weak intermolecular hydrogen bonds and hydrophobic interactions between the UIMs and Di-Ub (K-63 linked), thereby making the binding interactions unfavorable for ubiquitin. Since binding affinity of individual UIM for mono-ubiquitin is low [42], an avidity-based mechanism probably makes the interaction between RAP80 and Lys 63-linked polyubiquitin highly robust. Co-operative binding between multiple UIMs and ubiquitin chains likely occurs, which favors the interaction of second UIM with ubiquitin after positioning of the first [39]. It has been reported [30] that expression of RAP80 ΔE81 allele abates recruitment of BRCA1 complex at DSB site, which further augment chromosomal aberration (chromatic breaks). The results presented in this study also suggest that deletion of 81 Glutamic acid residue significantly obliterates RAP80 structure and impairs it's binding with polyubiquitin chain. Unstable nature of mutant and di-ubiquitin complex may be responsible for defective recruitment of RAP80-BRCA1 complex to the DNA damage sites. Defective DNA damage repair perhaps leads to chromosomal aberration as shown in the model (**Figure 6**). Prolific comparison of RAP80 ΔE81 with wild type will help in understanding its role in various diseases and repair defects. It will further explore the possibility of structure based inhibitor design for therapeutic application that can compensate the effect of such mutation.

## Materials and Methods

Molecular biology or analytical grade chemicals were purchased from Sigma-Aldrich, unless otherwise specified with more than 99.99% purity. Restriction enzymes were purchased from Fermentas.

### Gene cloning, protein expression and purification

Q96RL1 gene (1–390) in pGEFP vector (Kind gift from J. Chen) was PCR amplified (Thermocycler, Biorad) followed by restriction digestion (BamH1/EcoR1), T4-ligation and cloned into pGEX-kT (Kind gift from John A. A. Ladias) vector. Primers (Sigma-Aldrich) having a TEV protease cleavage site (E-N-L-Y-F-Q/S) were used for PCR amplification. Positive clones were selected by restriction digestion followed by DNA sequencing. c.241–243delGAA mutation was incorporated into wild type gene construct using site- directed -mutagenesis. PCR amplified product was digested with Dpn1 (Fermentas) and transformed into *E.coli* DH5α bacterial strain. Incorporation of desired mutation was confirmed by DNA sequencing. For protein expression and purification, vector construct was transformed into *E. coli* BL21 (DE3) cells (Novagen) and a single colony was inoculated in LB broth to obtained pre-inoculums culture. Protein was over-expressed in *E.coli* BL21 (DE3), and culture was grown at 37°C until O.D_600_ reached between 0.6–0.8, followed by induction with 0.4 mM IPTG at 18°C overnight. Harvested bacterial pellet was re-suspended in 10 mM HEPES buffer containing 300 mM NaCl, 5 mM BME, 0.1 mM EDTA and 5% ethylene glycol at pH 7.5 (HNBEEG buffer). Cells were disrupted by sonication (Branson Sonifier) and supernatant was collected after centrifugation. Soluble protein was passed through the pre equilibrated glutathione resin and then washed with HNBEEG buffer to remove impurities. Bound fusion protein was cleaved with TEV protease to elute the protein of interest. Protein was further passed through a gel filtration column (Superdex 200, GE) to remove aggregates, etc. and was analyzed using SDS-PAGE for purity.

### Protein Estimation

Quantification of RAP80 wild type and ΔE81 were performed with Bradford protein estimation protocol according to manufacturer's (expedon) instructions. Several dilutions of BSA were prepared as a standard reference. The absorbance was recorded in three sets at λ 595 nm using a spectrophotometer (Shimadzu). Average values were considered, and concentration of sample was determined by intra-plotation of BSA standard curve [43] [44].

### Molecular Modeling and docking

Protein structures RAP80 (ΔE81UIMs, 79–124 amino acids) was modeled using homology modeling server considering NMR structure (PDB ID; 2RR9) as template. Good-quality models were selected based on overall stereo chemistry, and validated using Ramachandaran plot and protein structure validation server “**SAVES**” (Metaserver for analyzing and validating protein structures, http://nihserver.mbi.ucla.edu/SAVES/). SAVES mainly comprises five programs, Procheck, What_check, Errat, Varify_3D and Prove. Modeled structure was simulated for 5 ns using Desmond software (Schrodinger) and superimposed on wild type complex. PDBsum was obtained to analyze the interactions.

### Limited proteolysis

Equal concentration of RAP80 wild type and ΔE81 (0.2 mg/ml) was incubated with Trypsin and chymotrypsin separately so that final concentrations of proteases were 40 ρg/µl and 10 ρg/µl respectively. Reaction mixture was incubated for different time 0, 10, 30, 60, 180, 360 minutes at 37°C (trypsin) and 25°C (chymotrypsin), respectively. Reaction was terminated individually by adding 1 mM PMSF (sigma-Aldrich). Samples were heated by adding equal volume of laemmli buffer and analyzed by SDS-PAGE. This experiment was performed in three sets with control which was untreated with proteases [45] [46].

### Surface Plasmon Resonance

Interaction studies between RAP80 wild type, ΔE81 and di-Ub (K63 linked) were performed using BIAcore 3000 (GE). A total of 5 µg ligand (Di-Ub K-63 linked) was immobilized on CM5 sensor chip using amide coupling method. Different concentration (0,100, 200, 400, 800, 1600 nM) of RAP80 wild type and ΔE81 (analytes) were passed on the chip at a flow rate of 20 µl/min. Interaction was quantified in terms of Response unit (RU). Sensor chip was regenerated with 2 M glycine pH 2.0. Sansogram was obtained after blank correction. The experiment was repeated thrice.

### GST pull down assay

Bacterial pellet of GST-RAP80 wild type and ΔE81 were re-suspended in HNBEEG buffer and sonicated. Soluble fusion protein(s) bound on glutathione resin (0.5 mg/ml) was used to capture prey {Di-Ub (K-63 linked) 10 µg, Boston Biochem}. Resin was pre-equilibrated with same buffer and loaded on SDS-PAGE. Complex was transferred to PVDF membrane (Millipore) and was probed with anti-ubiquitin antibody (Abcam). The experiment was repeated thrice by taking GST as control.

### Circular Dichroism

Far-UV CD spectrum were recorded using a Circular Dichroism (CD) polarimeter (Jasco J-810, Japan). 10 µM protein (in 2.5 mM HEPES pH 7.5, 50 mM NaCl) was scanned in a wavelength range of 200–240 nm at 10°C. Average blank corrected data of three independent scans were considered. Molar ellipticity was calculated, and data analysis was done using DichroWeb server (http://dichroweb.cryst.bbk.ac.uk) [47] [48] [49] [50] [51]. For thermal denaturation, wild type and ΔE81 protein (10 µM) were unfolded in a temperature range of 10–60°C at 218 nm wavelength. Fraction unfolded was calculated at the different temperatures. The experiment was performed three times independently, and an average data was considered. Data fitting was done according to two-state transition model, and thermodynamic parameters were calculated.

### ANS Fluorescence spectroscopy

The ANS (1-Anilino-8-Naphthalene Sulfonate) fluorescence was monitored using a Fluorescence spectrophotometer (Horiba, USA) at an excitation wavelength of 360 nm. For thermal denaturation, 2 µM protein (wild type and ΔE81) was incubated with 10 µM ANS for 10 min and emission scans were recorded from wavelength 400–600 nm in a temperature range of 5–60°C. Thermodynamic parameters were obtained by curve fitting according to two-state transition models [52]. These experiments were performed three times independently, and average blank corrected data was considered for curve fitting in two-state transition models [53]

### Differential Scanning Calorimetry

Thermal unfolding of wild type and ΔE81 was done using Differential Scanning Calorimetry (Setaram μDSC3 evo, USA). Protein and buffer were filtered and degassed prior to the scan. A total of 2 mg protein (RAP80 wild type) and 0.2 mg (ΔE81) in solution form was allowed to unfold in 5–60°C temperature range with a temperature increment rate of 1°C/min. The experiment was repeated thrice independently. Data was fitted locally by “CALISTO” software according to two-state transition model. The thermodynamic reversibility of the protein unfolding was determined by heating the sample just above the transition maximum, cooling instantaneously, and then reheating. Thermal denaturation transitions were found irreversible due to absence of transition(s) in second run.
